# Growth-associated polyhydroxybutyrate accumulation in *Azospira suillum* PS during aerobic and perchlorate respiration

**DOI:** 10.3389/fmicb.2026.1744475

**Published:** 2026-03-19

**Authors:** David A. O. Meier, Benjamin Glazer, V. Celeste Lanclos, Hans K. Carlson, John D. Coates

**Affiliations:** 1 Department of Plant and Microbial Biology, University of California, Berkeley, Berkeley, CA, United States; 2 Environmental Genomics and Systems Biology Division, Lawrence Berkeley National Laboratory, Berkeley, CA, United States

**Keywords:** *Azospira suillum* PS, growth associated carbon storage, metabolism, perchlorate reducing bacteria, polyhydroxybutyrate

## Abstract

Polyhydroxyalkanoates (PHAs) are widespread microbial storage polymers increasingly recognized for roles beyond carbon and energy storage, including redox homeostasis and stress physiology. While PHA accumulation is classically associated with stationary-phase metabolism under severe nutrient imbalance, comparatively little is known about growth-associated PHA synthesis in facultative anaerobes with unusual respiratory strategies. Here, we investigated polyhydroxybutyrate (PHB) metabolism in *Azospira suillum* PS, a genetically tractable perchlorate-reducing bacterium capable of both aerobic and anaerobic respiration. Using physiological growth experiments, targeted gene deletions, PHB extractions, and intracellular redox measurements, we examined PHB accumulation under varying respiratory and nutrient conditions. We demonstrate that PHB accumulates during exponential growth under moderately nitrogen-limited conditions, both aerobically and during perchlorate respiration, representing a rare example of growth-associated PHB synthesis under anaerobic conditions. Genomic analysis revealed four *phaC* homologs, one of which could not be deleted under the experimental conditions tested and co-localized with *phaB* and *phaR*. Redox profiling further revealed a strong positive correlation between PHB accumulation and intracellular NADPH/NADP^+^ ratios. Together, these findings expand the physiological contexts in which PHB synthesis is known to occur and highlight perchlorate-respiring bacteria as underexplored model systems for studying growth-integrated carbon and redox storage strategies.

## Introduction

The use of processed polymers by humans dates to at least 1,600 B. C. E. (Hosler et al., 1999), but the invention of modern thermoplastics by Charles Goodyear in 1839 revolutionized their industrial and environmental impact. Today, thermoplastics are indispensable across nearly every sector due to their lightweight, durable, and versatile properties driving global plastic production to more than 380 million tons annually (Geyer et al., 2017). Because most conventional plastics are derived from fossil hydrocarbons and are not biodegradable, their accumulation in the environment has intensified interest in biologically derived, degradable alternatives. Among the biodegradable alternatives under investigation, polyhydroxyalkanoates (PHAs) have attracted sustained interest because they are synthesized naturally by microorganisms from renewable carbon sources, and compostable under all ambient conditions (Rosenboom et al., 2022).

PHA is intracellular microbial polyesters that function as reserves of carbon, energy, and reducing power (Williamson and Wilkinson, 1958). Among PHAs, polyhydroxybutyrate (PHB) is the best studied, having first been identified in *Bacillus subtilis* in 1926 (Lemoigne, 1926). Although PHB was originally characterized as an inert storage compound synthesized under conditions of nutrient imbalance, PHAs are now increasingly understood as dynamic participants in microbial physiology, contributing to redox balancing, stress mitigation, and metabolic flexibility across diverse environmental contexts (Senior et al., 1972; Obruca et al., 2021; Tang et al., 2022; Sander et al., 2024). Their widespread distribution among bacteria and archaea, including organisms occupying extreme and anaerobic niches, suggests that PHAs play roles extending well beyond carbon storage during stationary phase (Emeruwa and Hawirko, 1973; Nanninga and Gottschal, 1986; Amos and McInerney, 1989; Poli et al., 2011).

In most well-characterized systems, PHB, the most common short-chain-length PHA (scl-PHA), accumulates during stationary phase in response to pronounced nutrient limitation, typically excess carbon relative to nitrogen, phosphorus, or oxygen. This paradigm has shaped both physiological and applied studies of PHB metabolism and is reinforced by work in canonical model organisms. For example, in the best-studied PHB-producing bacterium, *Cupriavidus necator*, polymer accumulation is robust but typically decoupled from exponential growth, instead requiring nutrient limitation or process-level interventions such as batch or fed-batch cultivation (Henderson and Jones, 1997; Sander et al., 2024). The prevalence of such systems has contributed to prevailing assumptions regarding PHB as a late-stage storage polymer rather than a growth-integrated metabolic process.

In contrast, growth-associated PHB accumulation, defined as polymer synthesis concurrent with exponential biomass increase, remains comparatively rare (Senior et al., 1972; Page and Knosp, 1989; Ackermann and Babel, 1997; Cristea et al., 2018; Scott et al., 2021; Woo et al., 2024). When observed, growth-associated PHB synthesis challenges the classical view of PHB as a deferred storage compound and raises fundamental questions regarding its physiological function during active growth. Recent work has expanded the known diversity of PHB-producing organisms to include anaerobes, phototrophs, syntrophs, oligotrophs, and extremophiles, demonstrating that PHB synthesis can occur across a broad range of metabolic and respiratory regimes (Dawes and Senior, 1973; Nanninga and Gottschal, 1986; Amos and McInerney, 1989; Costa et al., 2018; Wang et al., 2019; Koller and Rittmann, 2022; Bennett et al., 2024). In several of these systems, PHB has been implicated in redox homeostasis, serving as a metabolic sink for excess reducing equivalents when electron acceptor availability fluctuates or when respiratory pathways are constrained. Together, these observations support the view that PHB synthesis may be tightly coupled to intracellular redox state rather than being driven solely by nutrient limitation.

PHB biosynthesis proceeds through a conserved core pathway encoded by *phaA* (*β*-ketothiolase), *phaB* (acetoacetyl-CoA reductase), and *phaC* (PHA synthase), which together convert acetyl-CoA–derived intermediates into polymerized 3-hydroxybutyryl-CoA. Additional regulatory and structural proteins, including *phaR* and granule-associated *phaP* (phasin), modulate polymer accumulation, granule number, and granule morphology (Pötter et al., 2005). Based on substrate specificity and subunit composition rather than evolutionary lineage, PHA synthases (*PhaC* enzymes) are grouped into four functional classes (I–IV). Classes I, III, and IV primarily synthesize scl-PHAs such as PHB but differ in enzyme architecture: Class I enzymes function as homodimers of identical *PhaC* subunits, whereas Classes III and IV form heterodimeric complexes with accessory subunits PhaE and PhaR, respectively. In contrast, Class II PHA synthases are specialized for medium-chain-length PHAs (mcl-PHAs) and typically involve multiple PhaC isoforms (e.g., PhaC1 and PhaC2) without accessory subunits such as PhaE or PhaR. A deeper understanding of how feedstock composition and PHA synthase structure influence polymer diversity and accumulation is needed.

Dissimilatory perchlorate-reducing bacteria (DPRB) represent a promising but understudied group in this context. Perchlorate (ClO₄^−^) is a highly oxidized, water-soluble oxyanion with a biological redox potential comparable to oxygen (E°′ = +0.797 V) (Youngblut et al., 2016). In DPRB, perchlorate is reduced to chlorite by the perchlorate reductase complex (PcrAB), followed by dismutation into chloride and molecular oxygen via chlorite dismutase (Cld), with the generated oxygen subsequently respired by the cell (Youngblut et al., 2016). This pathway enables endogenous O₂ generation under otherwise anoxic conditions, supporting oxygenase-dependent metabolism without external aeration (Carlström et al., 2015). The conserved and mobile perchlorate reduction island (PRI) allows this metabolism to be distributed across diverse taxa (Melnyk et al., 2014). To date, the only indication that DPRB may accumulate PHB comes from a single report in *Dechloromonas agitata* CKB, where electron-dense intracellular inclusions consistent with PHA granules were observed, but without genetic or biochemical confirmation (Bruce et al., 1999).

*Azospira suillum* PS is a facultative anaerobe and genetically tractable perchlorate-reducing bacterium that provides a useful system for examining how PHB metabolism integrates with respiratory flexibility and intracellular redox balance. In this study, we investigate the genetic and physiological basis of PHB accumulation in *A. suillum* PS during both aerobic and perchlorate-respiring growth. By combining comparative genomics, targeted gene deletions, polymer quantification, and redox profiling, we characterize growth-associated PHB synthesis and its relationship to intracellular redox state. These findings extend emerging models of PHB as a growth-integrated, redox-coupled metabolic process to perchlorate-respiring bacteria and establish *A. suillum* PS as a model for studying the physiological role of PHB in facultative anaerobic systems.

## Materials and methods

### Bacterial strains and plasmids

*Azospira suillum* PS (ATCC BAA-33/DSMZ 13638) was revived from laboratory freezer stocks and used as the wild-type strain for all genetic manipulations. *Escherichia coli* XL1-Blue was used for plasmid propagation and cloning. A full list of primers (Supplementary Table 1), strains (Supplementary Table 2), and plasmids (Supplementary Table 3) is provided in the Supplementary materials. Prior to all experiments, strains were streaked from freezer stocks to obtain single colonies.

### Mutant construction

Gene deletion mutants in *A. suillum* PS were generated using a sacB-based counterselection approach (Gay et al., 1983). Electrocompetent cells were transformed with either integrative (~500 ng) or replicative (~50 ng) plasmids in 1 mm electroporation cuvettes using a Gene Pulser Xcell electroporator (Bio-Rad, USA) with the following settings: 1.8 kV, 25 μF, and 200 *Ω*. Cells were chilled on ice before electroporation; cuvettes were kept at room temperature. After pulsing, cells were recovered in pre-warmed ALP medium at 37 °C for 6 h, then 100 μL of culture was plated directly on ALP agar containing 50 μg/mL kanamycin. Colonies appearing within 48 h were considered valid transformants and picked into 500 μL ALP broth with 50 μg/mL kanamycin. After 4 h of growth, cultures were plated on ALP agar with 6% sucrose for counterselection. Representative colonies were screened by colony PCR to confirm deletions. Glycerol stocks were prepared at 15% final concentration for storage at −80 °C.

### Culture conditions and growth media

*E. coli* strains were cultured in LB medium with 50 μg/mL kanamycin. Wild-type and mutant *Azospira suillum* PS strains were grown in acetate minimal medium (AMM). One liter of AMM contained: 0.49 g sodium phosphate monobasic dihydrate, 0.97 g sodium phosphate dibasic anhydrous, 0.1 g KCl, 0.25 g NH₄Cl, and 10 mL each of vitamin and mineral mix (Bruce et al., 1999). The sodium acetate concentration in AMM was either 0.82 g/L acetate for 10 mM AMM or 2.05 g acetate for 25 mM AMM. To prepare mixed carbon source media (acetate, lactate, pyruvate), AMM was supplemented with 2.0 g yeast extract, 7.6 mL of a 60% (w/w) sodium lactate solution, and 1.10 g sodium pyruvate. For solid media, 15 g/L agar was added. *A. suillum* PS was cultured in 250 mL baffled Erlenmeyer flasks containing 50 mL of medium at 37 °C and 250 rpm. Optical density at 600 nm (OD₆₀₀) was measured using a 1 mm pathlength cuvette on a Genesys 20 spectrophotometer (Thermo Scientific, USA).

### PHA extraction and GC–MS analysis

PHB was quantified from 40 mL of culture. Samples were harvested by centrifugation at 7,197 × g for 10 min at 4 °C, and pellets were stored at −80 °C. Acidic methanolysis was performed as described by Juengert et al. (2018). Extracts were analyzed by gas chromatography–mass spectrometry (GC–MS) using a 7890A GC system (Agilent Technologies, USA) equipped with a DB-WAX UI column and coupled to a 5975C XL EI/CI Mass Selective Detector. The temperature program was: 80 °C for 2 min, ramp to 210 °C at 10 °C/min, then to 250 °C at 50 °C/min with a 1 min hold. Poly[(R)-3-hydroxybutyric acid] (Santa Cruz Biotechnology, USA) was used for the standard curve, and methyl benzoate (Sigma-Aldrich, USA) served as the internal standard. Compound identity was confirmed by total ion count (TIC) spectra matching the NIST EI database. The presence of polyhydroxyvalerate (PHV) and polyhydroxyhexanoate PHHX was assessed using a commercial PHA standard (Sigma-Aldrich, USA), and TIC peaks were compared with NIST spectra for 3-hydroxypentanoic acid methyl ester and 3-hydroxyhexanoic acid methyl ester.

### NAD(P)^+^/H quantification

Redox cofactor quantification was performed following the enzymatic cycling–based colorimetric assay described by Kern et al. (2014). Briefly, intracellular NAD(P)H and NAD(P)^+^ pools were differentially extracted using paired acid/base treatments to selectively destabilize oxidized versus reduced cofactors. Cell pellets were resuspended in either 0.2 M NaOH (to extract NAD(P)H) or 0.2 M HCl (to extract NAD(P)^+^), incubated at 50 °C for 10 min, neutralized with the reciprocal acid/base solution, and clarified by centrifugation.

Quantification was performed using an enzyme cycling reaction in which NAD(P)H drives reduction of phenazine ethosulfate (PES), which in turn reduces methylthiazolyldiphenyl-tetrazolium bromide (MTT). NADH was quantified using alcohol dehydrogenase and ethanol as substrate, while NADPH was quantified using glucose-6-phosphate dehydrogenase and glucose-6-phosphate. Ratios of reduced to oxidized pools were calculated from paired extracts. Samples were kept on ice and protected from light until incubated at 37 °C. To prevent redox degradation, only three samples were processed at a time. Quantification was performed using a Varian Cary 50 MPR Microplate Reader (Agilent Technologies, USA).

### Fluorescence microscopy

Microscopy was conducted as described by Juengert et al. (2018). Cells were harvested in exponential phase, washed once in PBS (8 g NaCl, 201 mg KCl, 1.42 g Na₂HPO₄, 245 mg KH₂PO₄ per liter, pH 7.4), and stained with 1 μg/mL Nile Red for 10 min in the dark. Samples were mounted on 1% agarose pads and visualized on a Zeiss LSM880 confocal microscope (Zeiss, Germany) using a 100 × Plan-Apochromat NA 1.4 objective. Nile Red was excited at 561 nm, and emission was detected from 570–620 nm using a GaAsP photon-counting detector. Differential interference contrast (DIC) images were acquired simultaneously. Image acquisition settings were consistent across all samples and processed in ZEN software v2.3 SP1 (Zeiss).

### Phylogenetic tree construction

Each *phaC* gene from *A. suillum* PS was queried against the NCBI nr database using BLASTP (Altschul et al., 1990). The top 250 unique hits (excluding “multispecies” entries) were retained per gene. PhaC homologs from *Azospira* species were manually reviewed and validated using HMMER v3.4 with TIGRfam-specific models (Finn et al., 2011). Alignments were generated with MUSCLE v3.8.31 and trimmed using trimAl v1.4 (Edgar, 2004; Capella-Gutiérrez et al., 2009). Phylogenies were inferred using IQ-TREE v2.0.6 with 1,000 ultrafast bootstraps and the Q.pfam+I + R7 model. Trees were visualized using iTOL v6 (Minh et al., 2020; Letunic and Bork, 2021).

### *In silico* comparative genomics

The genome of *A. suillum* PS was downloaded from NCBI (Sayers et al., 2024); *D. agitata* CKB and additional *Azospira* genomes were retrieved from JGI-IMG (Markowitz et al., 2006). Genomes were searched using HMMER v3.4 with a custom set of TIGRfams relevant to PHA metabolism (Supplementary Table 4). Presence/absence patterns of PHA biosynthesis genes were visualized using R v4.4.2 and RStudio v2024.12.0 + 467.

## Results

### Genomic and microscopic evidence for PHB granule formation in *A. suillum* PS and other perchlorate-reducing bacteria

**Figure 1:**
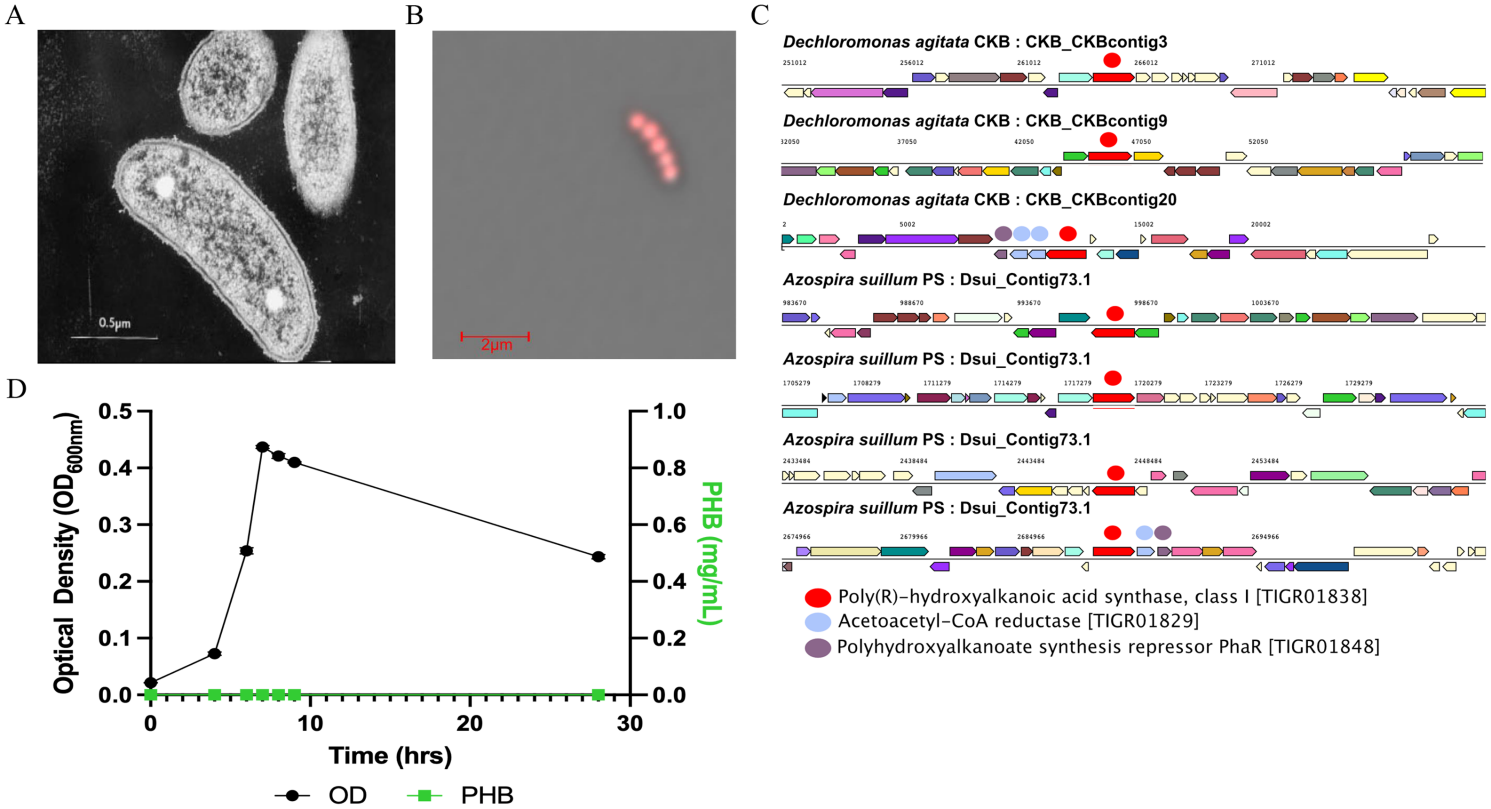
Microscopic and comparative genomic analysis of PHA production in *Dechloromonas agitata* CKB and *Azospira suillum* PS. **(A)** Transmission electron micrograph (TEM) of *D. agitata* CKB adapted from Bruce et al. (1999), showing electron-dense inclusions consistent with PHA granules. **(B)** Confocal fluorescence microscopy image of Nile Red-stained *A. suillum* PS grown under perchlorate-respiring conditions, overlaid with transmitted light, highlighting intracellular PHA granules. **(C)** Gene neighborhood analysis of *phaC* loci in CKB and PS, with neighboring PHA biosynthesis genes highlighted where present in the red box. **(D)** Growth (OD_600_) and absolute PHB production (mg/mL) by *A. suillum* PS during aerobic cultivation under nitrogen replete growth conditions.

Comparative genomic analysis of perchlorate-reducing bacteria revealed that both *D. agitata* CKB and *A. suillum* PS encode the complete set of genes required for PHA biosynthesis, including, phaC, and phaR (Figure 1C). Both genomes contain multiple phaC homologs, with three copies in CKB and four in PS, one of which in each organism is co-localized with phaB and phaR in an apparent operon arrangement. The remaining phaC copies are distributed elsewhere in the genome; only one is located adjacent to a phaP gene, and no additional cluster-level organization associated with PHA biosynthesis was observed. These genomic features motivated the selection of *A. suillum* PS as a genetically tractable system for experimentally characterizing PHA production under aerobic and perchlorate-respiring conditions.

To determine whether *A. suillum* PS produced PHB granules, we stained cultured cells with Nile Red, a lipophilic dye commonly used to visualize PHB granules and examined them using confocal microscopy (Figure 1B). Distinct intracellular lipid inclusions consistent with PHB granules were observed, with an approximate diameter of 0.32 *μ*m. Given the ~2 μm cell diameter of PS, the clustered granules span much of the intracellular space, corresponding to ~64% of the estimated cell volume being occupied by PHB granules. Cells stained positive for PHB granules under both aerobic and perchlorate-respiring conditions, providing the first clear evidence of PHA production linked to perchlorate respiration. This observation confirmed the genetic potential for PHB accumulation; however, it remained unclear whether polymer synthesis occurred constitutively or only in response to specific nutrient conditions.

**Figure 2:**
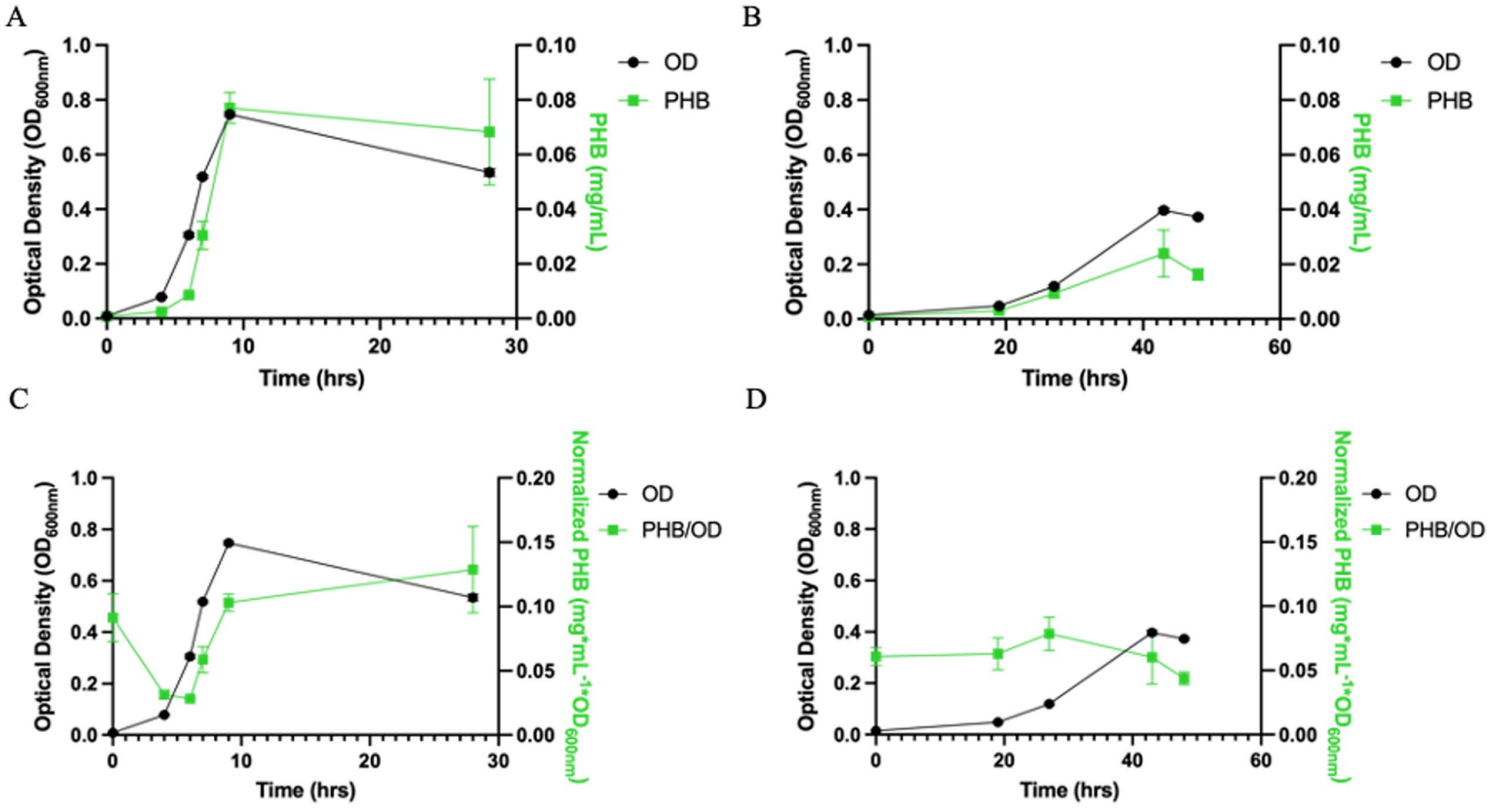
PHA production by *Azospira suillum* PS across electron acceptors with acetate as the electron donor under moderate nitrogen deplete conditions. **(A)** Growth (OD_600_) and absolute PHB production (mg/mL) during aerobic cultivation. **(B)** Growth (OD_600_) and absolute PHB production (mg/mL) during perchlorate-respiring growth. **(C)** Growth (OD_600_) and normalized PHB production (mg mL^−1^ OD_600_^−1^) under aerobic conditions. **(D)** Growth (OD_600_) and normalized PHB production (mg mL^−1^ OD_600_^−1^) during perchlorate respiration.

To establish whether PHB accumulation in *A. suillum* PS is constitutive or nutrient-dependent, we first evaluated growth under nitrogen-replete conditions. Cultures were grown in standard acetate minimal medium (AMM) containing 10 mM sodium acetate and 4.68 mM NH₄Cl, corresponding to a replete C:N ratio of approximately 1:0.23 relative to the canonical Redfield ratio (C:N = 1:0.15) (Redfield, 1934). Under these conditions, no detectable PHB was observed by GC–MS analysis (Figure 1D), confirming that PHB synthesis does not occur under nitrogen-replete growth. The culture exhibited a specific growth rate (*μ*) of 0.60 h^−1^ and a doubling time of 1.16 h, indicating robust biomass formation despite the absence of polymer accumulation. Although the final optical density under nitrogen-replete conditions (Figure 1) was lower than in carbon-enriched, nitrogen-limited cultures (Figure 2), this difference reflects the smaller amount of carbon supplied (10 mM vs. 25 mM acetate) rather than reduced growth capability. Consistent with this, the higher OD observed under the C:N = 1:0.094 condition results from increased carbon availability, not enhanced growth efficiency, as shown by the lower specific growth rate measured in that condition.

Given prior genomic and microscopic evidence suggesting PHB-producing potential, we next examined whether altering the carbon-to-nitrogen ratio could induce polymer synthesis. When the acetate concentration was increased to 25 mM while maintaining 4.68 mM NH₄Cl (C:N = 1:0.094), PHB production was observed under both aerobic and perchlorate-respiring conditions (Figures 2A–D). Under these carbon-enriched conditions, cell experience ~38% less nitrogen per unit carbon than under the canonical Redfield ratio, experiencing moderate nitrogen limitation. Under these conditions *A. suillum* PS exhibited a slightly reduced specific growth rate (μ = 0.50 h^−1^; doubling time = 1.39 h) but accumulated measurable intracellular PHB. These results demonstrate that PHB synthesis in *A. suillum* PS is triggered by a shift toward carbon surplus relative to nitrogen, rather than being a constitutive feature of its metabolism.

Following this finding, we conducted a detailed time-course analysis of PHB accumulation under the carbon-enriched condition to quantify the extent and dynamics of polymer synthesis during aerobic and perchlorate-respiring growth.

### Growth-associated PHB production in *Azospira suillum* PS under aerobic and perchlorate-respiring conditions

To quantify PHA production by *A. suillum* PS, GC–MS was performed on samples extracted via acidic methanolysis. In addition to quantifying total PHB produced by the culture, this method also enabled identification of the different monomers present. When PS was grown on acetate under either aerobic or perchlorate-respiring conditions 3-hydroxybutyric acid (3-HB) (the monomer of PHB) was detected. PHB was quantified across the growth curve and was found to be produced in a growth-associated manner (Figures 2A–D).

When grown aerobically, *A. suillum* PS reaches a maximum OD_600nm_ of 0.747 ± 0.008 during late exponential phase, at which point it had produced a maximum of 0.077 ± 0.006 mg/mL PHB providing a total yield of 0.10 g/L per OD_600nm_ (Figure 2A). Under perchlorate-respiring conditions with 10 mM perchlorate, PS reaches a lower maximum OD_600nm_ of 0.397 ± 0.009 and produces a maximum of 0.024 ± 0.008 mg/mL PHB or 0.06 g/L per OD_600nm_ (Figure 2B). When grown on perchlorate, PHB levels slightly decline as the culture enters stationary phase (Figure 2D). There is no significant reduction in PHB levels between peak and late-stage culture under aerobic conditions, despite a drop in OD_600nm_ of approximately 0.21. Based on the 1.6-fold higher PHB yield in aerobic versus perchlorate conditions, we further characterized PHB production by PS under aerobic growth. This growth-associated pattern under carbon-enriched conditions contrasts with the nitrogen-replete culture, where no PHB was detected, reinforcing that polymer synthesis in *A. suillum* PS is activated only when the C:N ratio exceeds a physiological threshold.

When examining normalized PHB levels relative to optical density, both aerobic and perchlorate-grown cultures show evidence of carryover PHB from the inoculum (Figures 2C,D). In aerobic cultures, this residual PHB is rapidly degraded during lag phase prior to new PHB accumulation during log phase, suggesting that the cells may utilize the carryover PHB as an additional electron donor alongside the supplied acetate during the initial phase of growth (Figure 2C). In contrast, perchlorate-respiring cultures do not exhibit this initial degradation phase; instead, normalized PHB/OD₆₀₀ remains stable during early growth and begins increasing only during exponential phase.

### Redox cofactor dynamics link NADPH availability to PHB biosynthesis in *Azospira suillum* PS

**Figure 3:**
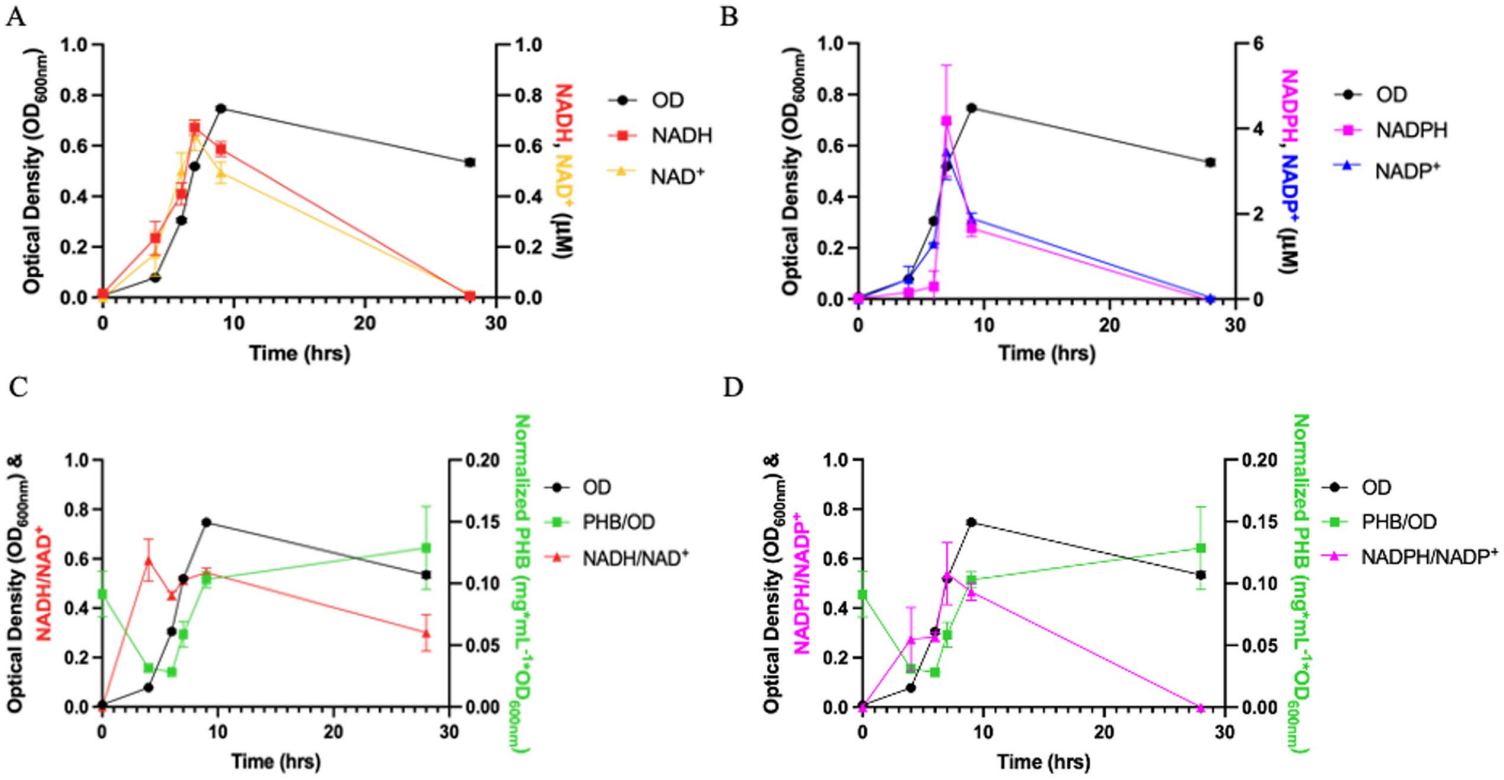
Normalized PHA production and intracellular redox state of *Azospira suillum* PS grown aerobically on acetate. **(A)** Growth (OD_600_) and absolute intracellular concentrations of NADH and NAD^+^ (μM). **(B)** Growth and absolute concentrations of NADPH and NADP^+^ (μM). **(C)** Growth, normalized PHB production (mg mL^−1^ OD_600_^−1^), and the NADH/NAD^+^ ratio. **(D)** Growth, normalized PHB production, and the NADPH/NADP^+^ ratio.

To assess the redox influence on PHB biosynthesis, we measured intracellular NADH/NAD^+^ and NADPH/NADP^+^ ratios over a 24-h culture period and compared these values to PHB accumulation (Figures 3A–D). When grown aerobically, *Azospira suillum* PS exhibited an average NADH/NAD^+^ ratio of 0.491—more than twice the reported aerobic values for *E. coli* (0.226) and *Klebsiella aerogenes* (0.195) (Wimpenny and Firth, 1972)—indicating a relatively reduced intracellular environment.

Spearman correlation analysis revealed a strong and statistically significant monotonic relationship between PHB levels and NADPH/NADP^+^ (*ρ* = 0.829, *p* = 0.042), while no significant correlation was observed with NADH/NAD^+^ (*ρ* = 0.600, *p* = 0.208). This is consistent with the known cofactor specificity of the PhaB acetoacetyl-CoA reductase enzyme. In *Cupriavidus necator* H16—a well-studied model organism for PHB production—this enzyme has been shown to preferentially use NADPH rather than NADH as the reducing equivalent source (Steinbüchel and Schlegel, 1991).

### Phylogenomic analysis reveals conserved, diversified *phaC* loci across the *Azospira* genus

**Figure 4:**
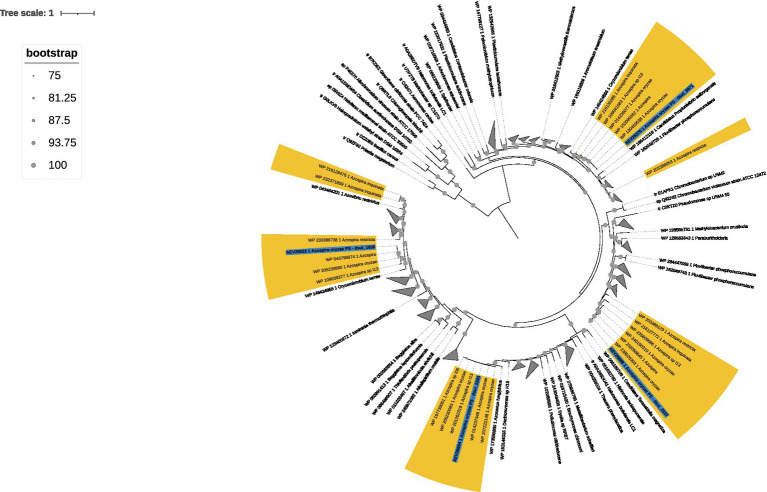
PhaC protein phylogeny within the genus *Azospira*. Maximum likelihood phylogenetic tree of PhaC protein sequences from members of the genus *Azospira*. Bootstrap support values (*n* = 1,000 replicates) are shown at each node. Branches corresponding to *Azospira* species are highlighted in yellow, and those from *A. suillum* PS are further annotated in green. Tip labels include the GenBank protein accession number followed by the corresponding NCBI organism name.

**Figure 5:**
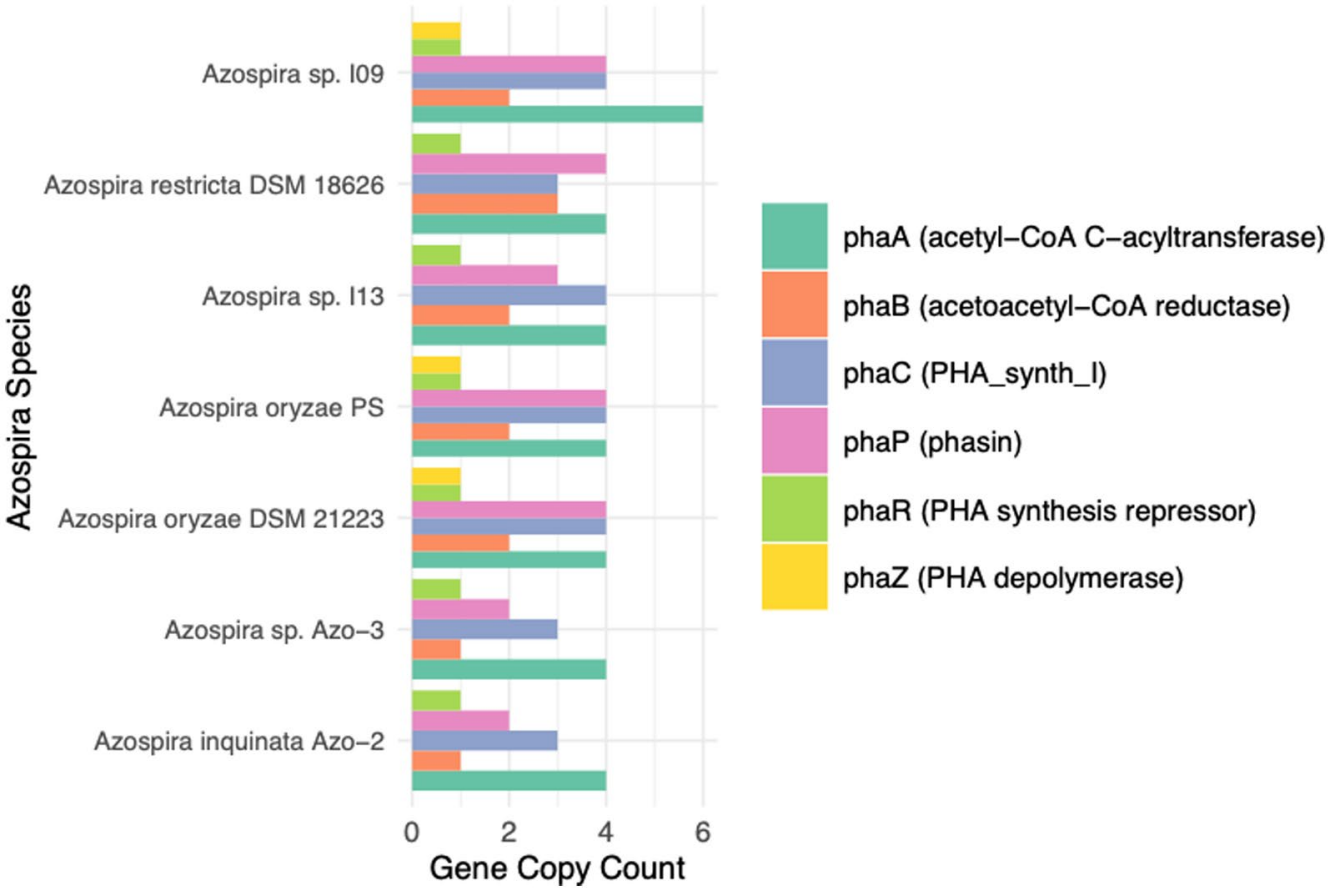
Stacked barplot showing the copy number of PHA-associated gene families identified in each available *Azospira* genome. Each bar represents a genome, and colored segments correspond to individual gene families detected by HMMER above noise cutoff thresholds. Values indicate total gene counts per family within each genome.

A maximum-likelihood phylogenetic tree (Figure 4) constructed from the four unique *phaC* gene products identified in *Azospira suillum* PS, along with their 250 closest homologs, revealed that each PhaC protein is nested within a monophyletic clade composed of *phaC* homologs from other *Azospira* species. Despite this shared genus-level affiliation, the four *phaC* variants from strain PS occupy distinct and divergent branches within the tree, suggesting that each *phaC* gene may have evolved independently. Furthermore, this analysis revealed that multiple *phaC* gene copies are consistently present in *Azospira* genomes. Comparative genomic analysis across the genus showed that each *Azospira* species encodes at least three distinct copies of *phaC*, alongside a single copy of the transcriptional regulator *phaR*, suggesting that PHA biosynthesis is a conserved metabolic feature within the genus (Figure 5). To assess whether these genes may have been acquired via horizontal gene transfer, we used IslandViewer (Bertelli et al., 2017), which predicts genomic islands based on sequence composition and comparative genomics. This analysis showed no evidence of horizontal gene transfer near the *phaC* loci in strain PS (data not shown).

**Figure 6:**
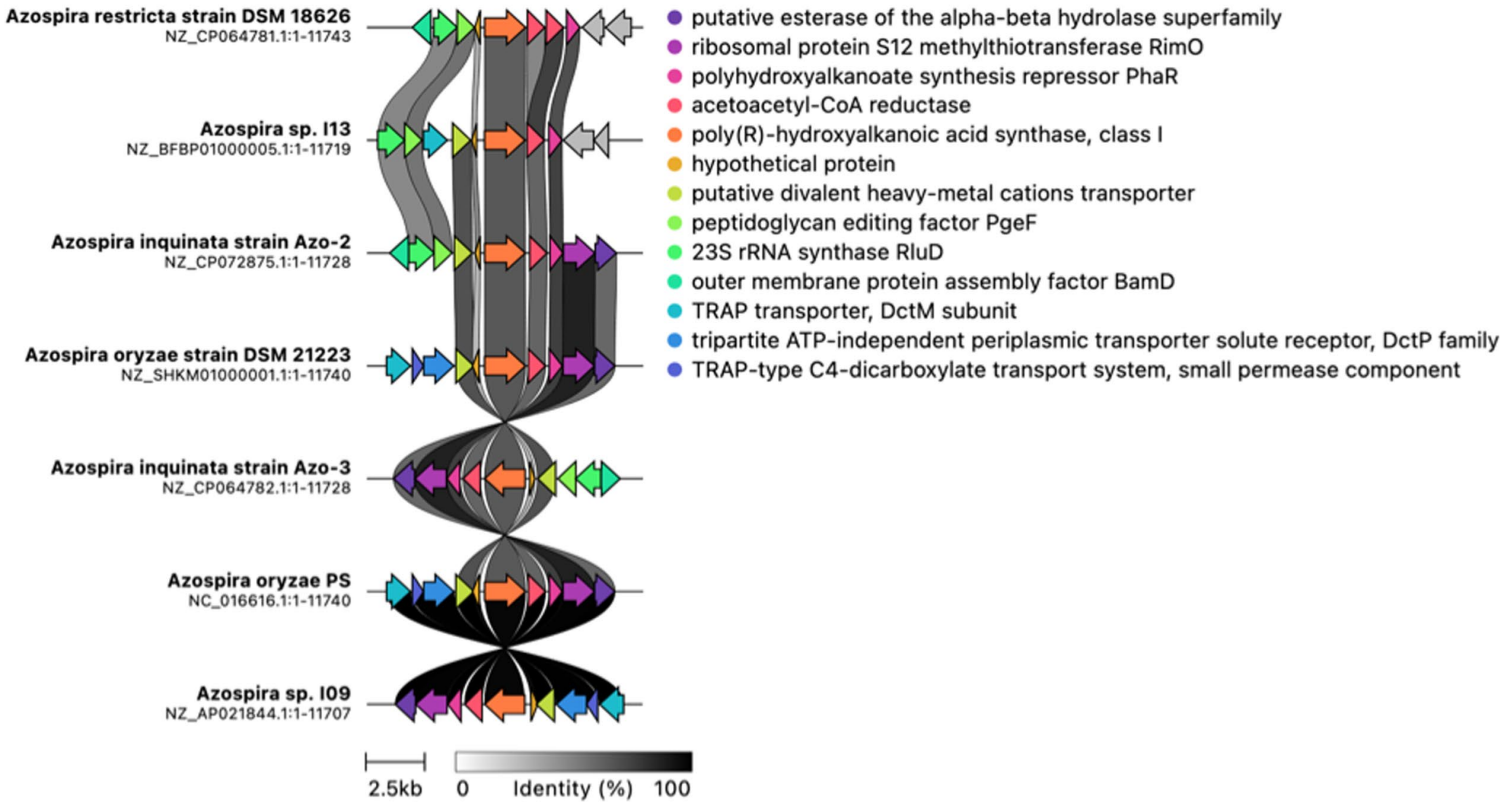
Nucleotide identity percentages for the *phaC* biosynthetic cluster co-localized with *phaB* and *phaR* across *Azospira* species, highlighting variation in conservation of this operon.

To assess conservation of gene neighborhoods, we used CAGE-CAT and Clinker to compare the genomic loci surrounding *phaC* across *Azospira* genomes (Gilchrist and Chooi, 2021). These tools align and visualize syntenic regions based on protein sequence identity and gene order, enabling rapid identification of conserved operons and structural rearrangements. This analysis (Figure 6) revealed that the local gene context of *phaC* is highly conserved, with pairwise amino acid identity of *phaC* homologs exceeding 70% across the genus, further supporting their functional importance in *Azospira*. However, while average nucleotide identity remains high across *Azospira* species, they do not share syntenic conservation of the broader PHA biosynthesis gene cluster.

### Functional characterization of *phaC* genes identifies phaC4 as non-deletable under tested conditions

Our analysis found that *Azospira suillum* PS encodes only class I *phaC* genes, which are typically associated with the production of scl-PHAs such as PHB. To investigate the four *phaC* genes in *A. suillum* PS responsible for PHA production, we attempted to generate deletion mutants for each gene. Knockout strains were successfully constructed for three out of the four *phaC* genes. However, under all tested growth conditions, we were unable to generate a mutant lacking the *phaC* gene with the locus tag *dsui_2537* (*phaC*4). Notably, this gene is co-localized with *phaB* and *phaR*. Previous transposon mutagenesis studies in strain PS also identified *phaC*4 as conditionally essential under all conditions tested (Supplementary Figure 1).

To further assess the functional role of each *phaC*, we compared the PHA monomer profiles of all knockout strains under both defined and complex media conditions. As shown in Table 1, none of the knockout strains displayed a PHA profile distinct from wild type. All strains consistently produced PHB, while PHV and PHHX were absent (data not shown). These results suggest that the conditionally essential *phaC*4 is playing an important physiological role during normal growth in nutrient replete media.

**Table 1:** Polyhydroxyalkanoate methyl esters produced by *Azospira suillum* PS when grown on complex or defined media.

Strains	PHB	PHV	PHHX
WT	+	−	−
*ΔphaC1*	+	−	−
*ΔphaC2*	+	−	−
*ΔphaC3*	+	−	−
*Δ(phaC1-phaC2)*	+	−	−
*Δ(phaC1-phaC3)*	+	−	−
*Δ(phaC2-phaC3)*	+	−	−
*Δ(phaC1-phaC2-phaC3)*	+	−	−

Summary of PHA monomers identified in *A. suillum* PS when cultivated on complex media (acetate, lactate, pyruvate with yeast extract) or defined acetate minimal medium. PS locus tags corresponding to each *phaC* gene are listed in Supplementary Table 5.

## Discussion

The central finding of this study is that PHB synthesis in *Azospira suillum* PS is growth-associated and occurs under moderate nitrogen limitation during both aerobic and perchlorate-respiring metabolism. Rather than accumulating as a late-stage storage polymer following growth arrest, PHB in PS is synthesized concurrently with biomass increase and remains quantitatively modest. This pattern suggests that PHB functions as a dynamic metabolic intermediate integrated into central metabolism rather than as a long-term carbon reserve.

The induction of PHB exclusively under carbon-enriched conditions indicates that polymer synthesis in PS is regulated by C:N imbalance rather than constitutive expression. Notably, increasing the severity of nitrogen limitation did not further enhance PHB accumulation, suggesting that once a carbon-surplus threshold is reached, additional nitrogen restriction does not proportionally stimulate polymer production. A similar pattern has been observed in other anaerobic organisms, including *Syntrophomonas wolfei*, in which PHB synthesis occurred independently of ammonium chloride concentration (Amos and McInerney, 1989). Importantly, PHB accumulation was observed under both oxygen-respiring and perchlorate-respiring conditions, indicating that this regulatory architecture is independent of terminal electron acceptor identity. This respiratory-mode agnosticism supports the interpretation that PHB flux in PS is more closely linked to intracellular redox balance and carbon partitioning than to the specific respiratory pathway in use.

Growth-associated PHB synthesis has been reported in a limited number of systems (Senior et al., 1972; Page and Knosp, 1989; Ackermann and Babel, 1997; Cristea et al., 2018; Scott et al., 2021; Woo et al., 2024), yet it remains comparatively uncommon relative to the classical paradigm in which PHB accumulates predominantly during stationary phase under pronounced nutrient imbalance (Anderson and Dawes, 1990; Sander et al., 2024). The phenotype observed in *A. suillum* PS therefore aligns with a smaller but growing body of literature suggesting that PHB can function as a growth-integrated metabolic sink rather than solely as a late-stage storage polymer.

Temporal analysis revealed distinct regulatory patterns between respiratory modes. Under aerobic growth, normalized PHB/OD₆₀₀ declined during lag phase, likely reflecting mobilization of stored carbon and reducing equivalents inherited from the inoculum, followed by renewed PHB accumulation during exponential growth. In contrast, perchlorate-respiring cultures did not exhibit this early degradation phase; PHB levels remained stable during early growth and increased only during exponential phase.

Using established correlations between OD₆₀₀ and cell dry weight for morphologically similar organisms (Glazyrina et al., 2010), the maximum aerobic PHB concentration in *Azospira suillum* PS corresponds to approximately 2.6–3.2% PHB per CDW, while perchlorate-respiring cultures accumulated approximately 1.5–2.5% PHB per CDW. Thus, PS accumulates measurable PHB under both respiratory modes, with aerobic growth supporting ~1.6-fold greater PHB per unit biomass. These modest PHB fractions stand in marked contrast to the large stationary-phase PHB reserves characteristic of classical storage organisms such as *Cupriavidus necator*, which can accumulate 60–80% CDW PHB under severe nutrient imbalance (Zhang et al., 2022). In PS, however, PHB is synthesized exclusively during active growth and remains quantitatively small, supporting the interpretation that this organism utilizes PHB as a dynamic metabolic intermediate rather than a long-term carbon store.

These PHB fractions fall within the quantitative range reported for other anaerobic microorganisms exhibiting growth-associated or redox-linked PHB accumulation, such as *Syntrophomonas wolfei* (~35 μg mL^−1^ PHB) (Amos and McInerney, 1989). However, they are lower than the PHB levels reported for moderately accumulating fermentative organisms such as *Clostridium botulinum* (9–13% CDW) (Emeruwa and Hawirko, 1973), and substantially below the large PHB reserves observed in strongly carbon-imbalanced anaerobes, including sulfate-reducing bacteria (20–43% CDW) (Hai et al., 2004). Comparable low-to-moderate PHB fractions have also been reported for some halophilic archaea, where PHB content spans a broad range (0.05–63% CDW) depending on species and carbon source (Poli et al., 2011).

The distinction between PS and *C. necator* is therefore not merely quantitative but fundamentally physiological. Whereas *C. necator* accumulates extraordinarily high PHB fractions only after entering stationary phase and only under severe nutrient imbalance, PS produces small but consistent quantities of PHB throughout exponential growth under both aerobic and perchlorate-respiring conditions. This growth-associated phenotype which is agnostic of respiratory-mode suggests a regulatory role for PHB linked to central metabolism and electron balance rather than carbon storage.

A strong and statistically significant correlation was observed between intracellular NADPH/NADP^+^ ratios and PHB content (Spearman *ρ* = 0.829, *p* = 0.042), implicating NADPH availability as a potential driver of PHB accumulation. Although this correlation suggests a role for NADPH in fueling PHB biosynthesis, it does not establish direct cofactor specificity of the biosynthetic enzymes. NADH and NADPH pools are often tightly coupled and metabolically interconvertible, and the observed association may therefore reflect broader intracellular redox state rather than exclusive NADPH usage. Nevertheless, this relationship is consistent with the known NADPH preference of PhaB acetoacetyl-CoA reductases in other organisms (Steinbüchel and Schlegel, 1991; Ling et al., 2018). Further biochemical characterization will be required to determine whether PhaB or related enzymes in PS preferentially utilize NADPH over NADH and to define how NADPH-generating reactions interface with PHB flux.

The elevated NADH/NAD^+^ ratios observed during aerobic growth in PS further support a relatively reduced intracellular environment compared to several well-characterized facultative bacteria (Wimpenny and Firth, 1972). Only respiratory generalists such as *Pseudomonas aeruginosa* have been reported to exhibit similarly high aerobic NADH/NAD^+^ ratios. While *Escherichia coli* lacks native PHA biosynthetic capacity, certain Pseudomonas species are capable of PHB production, although typically not in a growth-associated manner (Bertrand et al., 1990). The comparatively reduced redox state of PS may therefore create metabolic conditions that favor diversion of carbon flux into PHB as a mechanism for dissipating excess reducing equivalents.

Genomic analysis revealed four *phaC* genes encoding class I PHA synthases in *A. suillum* PS. Among these, *phaC4* is notable for its synteny with *phaB* and *phaR*, consistent with a conserved operon structure. No evidence of horizontal gene transfer was detected near any *phaC* locus, indicating that these genes are native and vertically inherited. Repeated attempts to generate a deletion mutant of phaC4 were unsuccessful under all growth conditions tested, whereas deletion mutants were readily obtained for the other three phaC genes. Independent random barcode transposon mutagenesis data in strain PS also failed to recover insertions in phaC4 (Supplementary Figure 1), demonstrating conditional essentiality under all conditions tested. Together, these findings indicate that PHB biosynthesis is deeply integrated into the core physiology of PS, potentially functioning as a mechanism for maintaining intracellular redox balance rather than serving solely as a dispensable storage pathway.

By combining genomic analysis, targeted gene deletions, polymer quantification, and redox profiling, this study establishes *Azospira suillum* PS as the first isolated perchlorate-reducing bacterium demonstrated to synthesize PHB and as a mechanistically tractable model for studying growth-integrated, redox-coupled PHB metabolism in facultative anaerobes. PHB synthesis was absent under nitrogen-replete conditions and was induced only when the carbon-to-nitrogen ratio was altered to impose an approximately 40% reduction in nitrogen availability relative to the canonical Redfield ratio, demonstrating that polymer accumulation is triggered by moderate nutrient imbalance rather than severe starvation. The induction of PHB exclusively under carbon-surplus conditions is consistent with classical C:N-governed regulation of PHB biosynthesis (Sander et al., 2024), while the strong linkage observed here between PHB accumulation, intracellular NADPH/NADP^+^ ratios, and phaCBR operon organization supports a model in which PHB flux is integrated with central redox metabolism.

Beyond defining a new physiological phenotype in perchlorate-respiring bacteria, these findings broaden the recognized metabolic contexts for PHA production and underscore the importance of examining PHB biosynthesis within its native metabolic and ecological framework. The growth-associated synthesis observed under both perchlorate-respiring and aerobic conditions provides a foundation for future biochemical and genetic studies aimed at dissecting regulatory control, enzyme specificity, and flux partitioning, and may inform exploration of redox-balanced or continuous cultivation strategies. However, such efforts will ultimately depend on a deeper mechanistic understanding of how NADPH metabolism, phaC paralog function, and redox homeostasis collectively govern PHB flux in this system.

## Data Availability

The datasets presented in this study can be found in online repositories. The names of the repository/repositories and accession number(s) can be found in the article/Supplementary material.
