# Supplementary material for: Growth-associated polyhydroxybutyrate accumulation in Azospira suillum PS during aerobic and perchlorate respiration
Source: Front Microbiol. 2026 Mar 19;17:1744475. doi: 10.3389/fmicb.2026.1744475 (PMC13044030; doi:10.3389/fmicb.2026.1744475)
Supplement: Supplementary file 2 [file Data_Sheet_1.docx]

Supplementary Material for Manuscript:

Bioplastic Production Potential of *Azospira suillum* PS: Growth-Associated PHB Production Under Aerobic and Anaerobic Conditions

David A.O. Meier^1^, Benjamin Glazer^1^, V. Celeste Lanclos^1^, Hans K. Carlson^2^, John D. Coates^1*^

^1^ Department of Plant and Microbial Biology, University of California, Berkeley, Berkeley, CA, United States

^2^ Environmental Genomics and Systems Biology Division, Lawrence Berkeley National Laboratory, Berkeley, CA, United States

## Supplementary Figures:

**Supplementary Figure 1 |** Transposon sequencing barcode data showing fitness of insertion mutants in the *phaCBR* gene cluster of *Azospira suillum* PS. The essentiality of *phaC* (dsui_2537) is supported by the absence of viable transposon insertions across growth conditions.


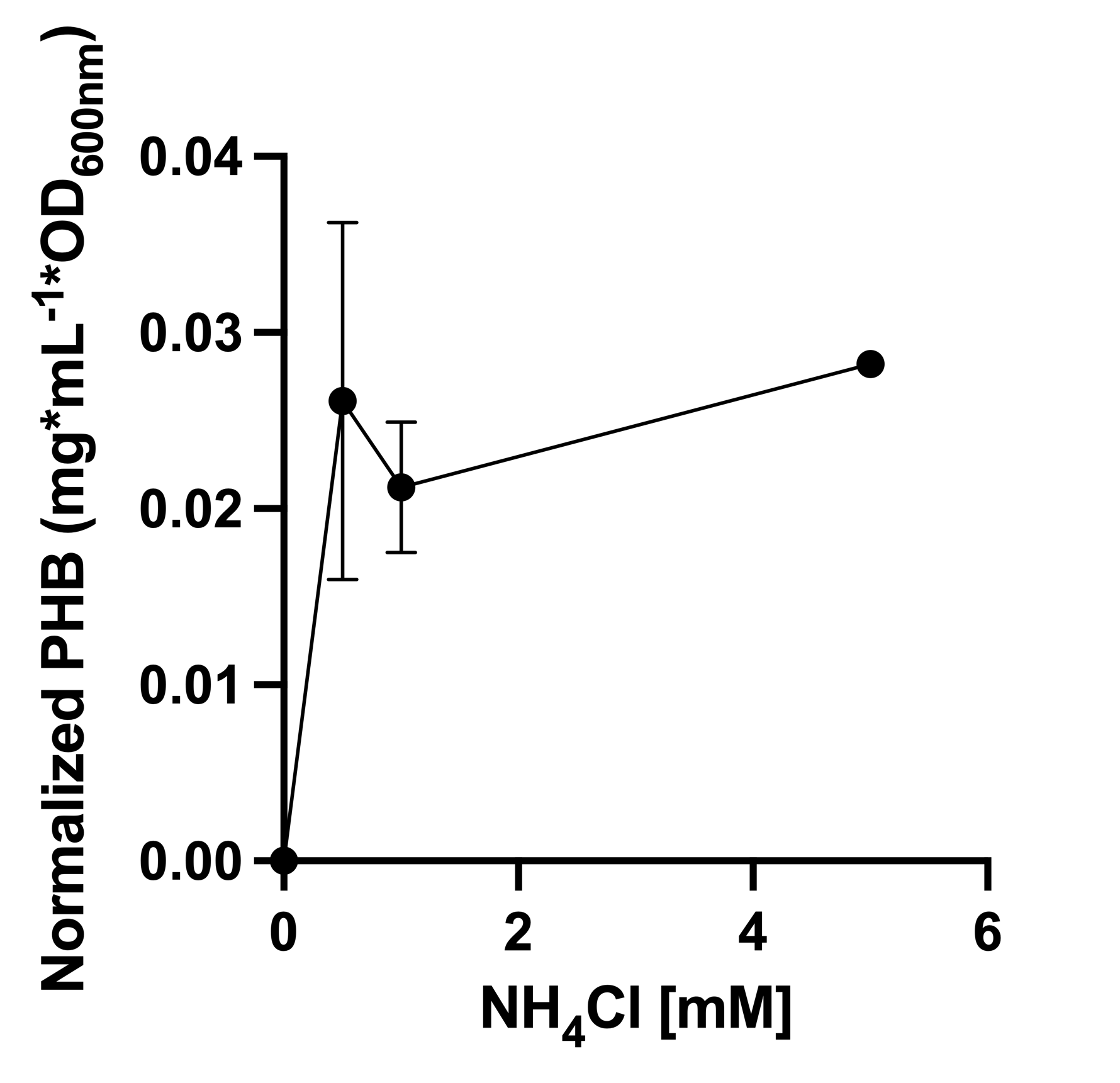


**Supplementary Figure 2 |** Effect of ammonium chloride concentration on normalized PHB production by *Azospira suillum* PS grown aerobically on 25 mM acetate. Samples were collected during early exponential phase.

**Supplementary Figure 3 |** GC-MS spectra of a commercial mixed PHA standard, confirming the presence of all tested monomers from a validated source.

**Supplementary Tables:**

**Table S1 |** Primer List

**Table S2 |** Strain List

**Table S3 |** Plasmid List

**Table S4 |** PHA TIGRfams

**Table S5 |** PS PHA Locus Tags
